# A rare case of a concomitant ovarian fibroma and malignant steroid cell tumor: insights into pathogenesis and steroidogenesis

**DOI:** 10.1186/s43046-025-00281-3

**Published:** 2025-05-19

**Authors:** Chihiro Inoue, Yuto Yamazaki, Hironobu Sasano, Yayoi Aoyama, Toyoharu Watanabe, Takashi Suzuki

**Affiliations:** 1 Department of Anatomic Pathology, Tohoku University Graduate School of Medicine, Sendai, Japan; 2 Department of Pathology, Tohoku University Hospital, Sendai, Japan; 3 Department of Gynecology and Obstetrics, Towada City Central Hospital, Towada, Japan

**Keywords:** Ovary, Sex cord-stromal tumor, Steroidogenesis, Pathology, Immunohistochemistry, Whole exome sequencing

## Abstract

**Background:**

Fibromas are common ovarian stromal tumors, while steroid cell tumors (SCTs) are rare, accounting for < 0.1% of ovarian neoplasms. Approximately, one-third of SCTs exhibit malignant behavior, but predicting malignancy remains challenging.

**Case presentation:**

A 73-year-old woman presented with nonspecific pelvic pain, and imaging revealed multiple pelvic masses. She underwent a simple hysterectomy and bilateral adnexectomy. Pathological examination revealed a unique colocalization of a fibroma and a SCT in the right ovary. One year later, the SCT recurred with lymph node metastasis. Morphological analysis and whole exome sequencing suggested a shared origin for the fibroma and SCT components. Notably, two missense mutations in *MUC4* were identified in the SCT, with immunohistochemistry confirming MUC4 overexpression. Steroidogenesis patterns in the SCT resembled those of adrenocortical carcinoma, indicating disorganized steroidogenesis and potentially explaining the absence of clinical endocrine abnormalities.

**Conclusion:**

This case underscores the rarity and complexity of concomitant ovarian fibroma and malignant SCT. The identification of *MUC4* mutations and disorganized steroidogenesis may provide insights into the pathogenesis of malignant SCTs. Further research is needed to understand the mechanisms and clinical implications of malignant SCT.

**Supplementary Information:**

The online version contains supplementary material available at 10.1186/s43046-025-00281-3.

## Background

Fibromas are the most common ovarian stromal tumors, accounting for 4% of all ovarian neoplasms. Steroid cell tumors (SCTs) are rare ovarian sex cord-stromal tumors, accounting for < 0.1% of all ovarian neoplasms, which comprise tumor cells with steroid-secreting morphology involving the ovarian parenchyma. Approximately, 50% of the patients with SCTs clinically present with androgenic symptoms, and 10% present with estrogenic symptoms; in rare cases, Cushing’s syndrome has been clinically reported. SCTs exhibit malignant behavior in approximately one-third of cases [1]. Pathological features such as a size > 7 cm, significant mitotic activity, necrosis, hemorrhage, and pronounced nuclear atypia have been reported to be associated with the malignant behavior of SCTs [2]. However, a recent report indicated that tumor necrosis, hemorrhage, and larger tumor size were significantly associated with International Federation of Gynecology and Obstetrics (FIGO) stage ≥ IB, reinforcing the conclusion that these features are not independent predictors of recurrence [3]. It is still difficult to predict malignant behavior based on pathological features, as the mechanisms by which SCTs acquire malignant characteristics remain unclear. Fibroma and SCTs are sex cord-stromal tumors, but there has been only one case report of an ovarian tumor comprising a fibroma and SCT, not otherwise specified (NOS) previously [4]. Here, we report the first case of an ovarian tumor comprising fibromas and a SCT, which was revealed to be malignant following metastasis 1 year after surgery.

## Case presentation

**Fig. 1:**
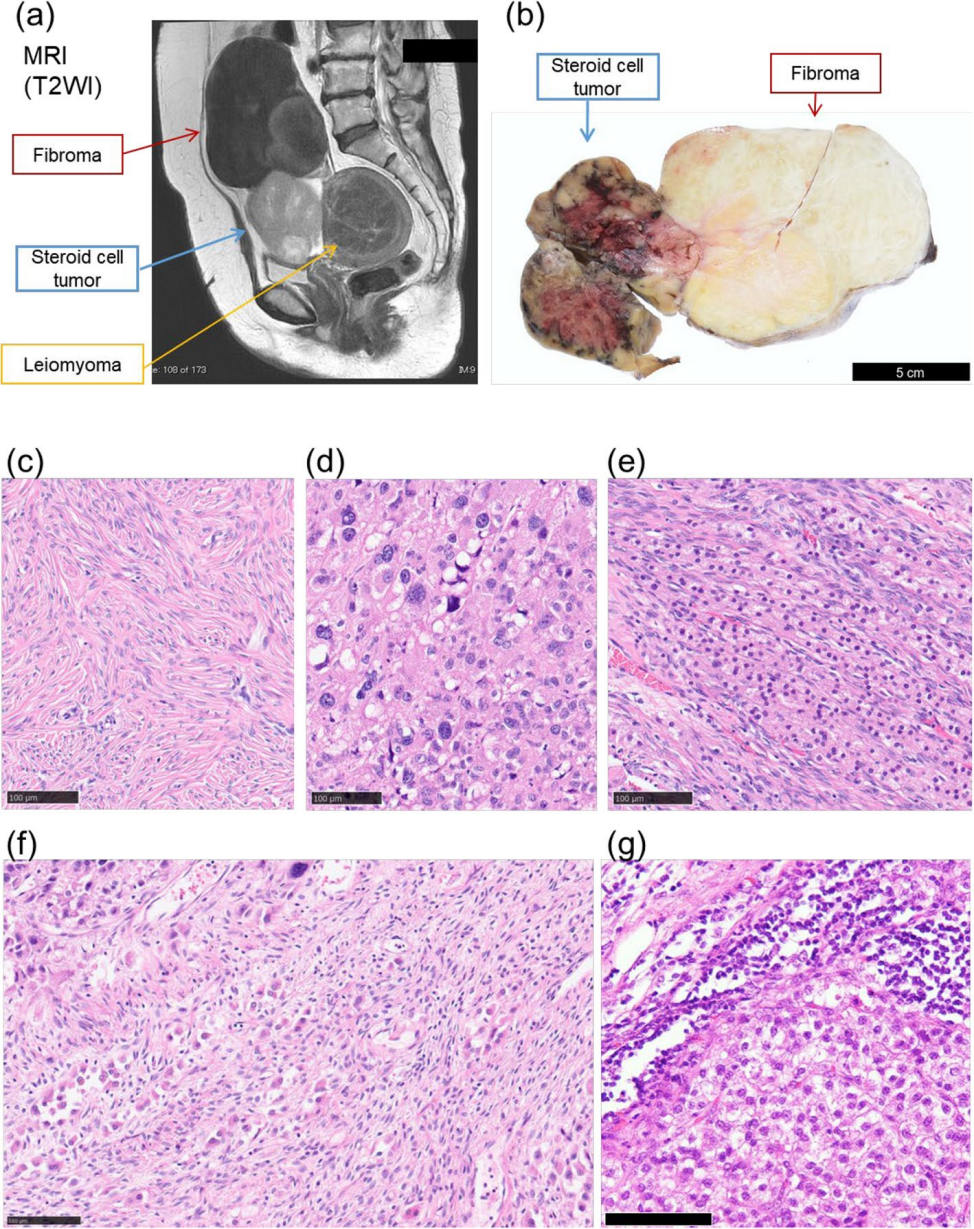
Radiological and pathological images of ovarian tumors. **a** Sagittal T2-weighted pelvic magnetic resonance imaging (MRI). The upper tumor with low signal intensity, middle tumor with high signal intensity, and right bottom tumor in the uterus is a fibroma, steroid cell tumor (SCT), and leiomyoma, respectively. **b** Macroscopically, the two tumors coexist. The fibroma is a white firm mass, and the SCT is a yellow tumor with bleeding and necrosis. Bar=5 cm. A high-power view of (**c**) fibroma and (**d**, **e**) SCT. **d** Most tumor cells show significant nuclear atypia. **e** Some tumor cells have small, round nuclei with less atypia. **f** Fibroma and SCT cells are intermingled in the boundary between the two tumor components. **g** Lymph node metastasis of the SCT. Metastatic tumor comprises steroid tumor cells with less atypia, similar to Figure 1e. **c**-**g** Bar=100 mm

A 73-year-old woman (gravida 2, para 2) presented with nonspecific pelvic pain. Ultrasonography revealed multiple pelvic masses, and she was referred to Towada City Central Hospital for further evaluation. Pelvic T2-weighted magnetic resonance imaging (MRI) demonstrated two extrauterine tumors adjacent to the anterior aspect of the uterine fundus: one with low signal intensity measuring 14 cm in diameter and another with high signal intensity measuring 9 cm in diameter (Fig. 1a). The low signal intensity tumor was suspected to be a fibroma, fibrothecoma, or benign Brenner tumor. In contrast, the high signal intensity tumor was clinically considered malignant due to its increased size compared to findings from contrast-enhanced CT performed 1 year earlier during a pacemaker evaluation for atrioventricular block treatment. A simple hysterectomy and bilateral adnexectomy were subsequently performed for treatment and diagnosis.

Macroscopically, the right ovarian mass comprised two different components: one with a white cut surface and stiff appearance, measuring 13 cm in diameter, and the other with a yellow cut surface and soft, measuring 9.5 cm in diameter, associated with intra-tumoral necrosis and hemorrhage (Fig. 1b). The two tumors were well circumscribed. A compressed nonneoplastic right ovary is detected on the surface of the white tumor. Additionally, multiple myomas were detected in the uterine body. No abnormalities were observed in the left adnexa.

Histologically, the white tumor comprised intersecting fascicles of spindle cells with bland spindle-to-ovoid nuclei and scant eosinophilic cytoplasm within a variably collagenous stroma and was subsequently diagnosed as a fibroma (Fig. 1c). The yellow tumor comprised diffuse proliferation of granular eosinophilic polygonal cells. The tumor cells showed significant nuclear atypia with large or multinucleated nuclei of irregular sizes and shapes (Fig. 1d). Some tumor cells with less atypia, small polygonal cells, and small round nuclei were predominantly observed in the peripheral area of the tumor (Fig. 1e). Mitotic activity was high (5 mitoses/10 high-power fields), with coagulative necrosis of tumor cells and intra-tumoral hemorrhage. No Reinke crystals were detected in the tumor cells. The tumor cells were immunopositive for inhibin alpha, calretinin, melan-A, and SF-1 and negative for pan-cytokeratin (AE1/AE3) and FOXL2 (Table 1). The tumor was diagnosed as a SCT-NOS based on morphology and immunohistochemistry results. The right ovarian mass comprised ovarian fibroma and SCT components. SCT cells with less atypia were observed, intermingling with fibroma cells in the boundary area between the two tumor components (Fig. 1f).

**Table 1:** Immunohistochemistry results

		Primary site		Metastatic site
Antibody	SCT cells with significant atypia	SCT cells with less atypia	Fibroma cells	SCT cells
pan-CK (AE1/AE3)	-	-	-	(Not examined)
α-Inhibin	+	+	-	+
Melan-A	+>weak+>-	+>-	-	+/weak+/-
Calretinin	+	+	->>weak+	+
FOXL2	-	-	+	-
SF-1	+	+	+>>-	+
StAR	+>-	+	-	+
SCC	Weak+>>-	+>weak+	-	+
3βHSD	Weak+>-	+>weak+	Weak+	Very weak+
c17	+/weak+/-	+	->>very weak+	+
c21	->>+	-	-	-
CYP11B1	Weak+>+	->>weak+	-	->weak+>>+
CYP11B2	-	-	-	-
17βHSD1	Weak+>+	+>weak+	+	Weak+>>+
17βHSD2	-	->weak+	Weak+>-	-
Aromatase	Weak+>->+	+>->weak+	->very weak+	Weak+>+
5α1	+>>-	+>weak+	Weak+	Weak+>+>-
5α2	-	-	-	-
EST	->>>weak+	Very weak+	-	-
STS	-	Weak+>+	->>weak+	->weak+
DHEA-ST	+>weak+>-	+	-	+>weak+>-

Immunohistochemical expression was evaluated using the PRIME notation method [5]

SCTs have all the factors that predict malignant behavior, including size > 7 cm, significant mitotic activity, necrosis, hemorrhage, and significant nuclear atypia [2]. Therefore, the patient received six cycles of paclitaxel-carboplatin postoperatively; however, the SCT recurred with para-aortic lymph node metastasis 1 year postoperatively. The recurrent tumor was surgically resected and found to comprise monotonous steroid cells with less nuclear atypia (Fig. 1g). No additional chemotherapy was administered because of recurrence. No other recurrent lesions were detected on the follow-up CT 3 months after the second surgery.

**Fig. 2:**
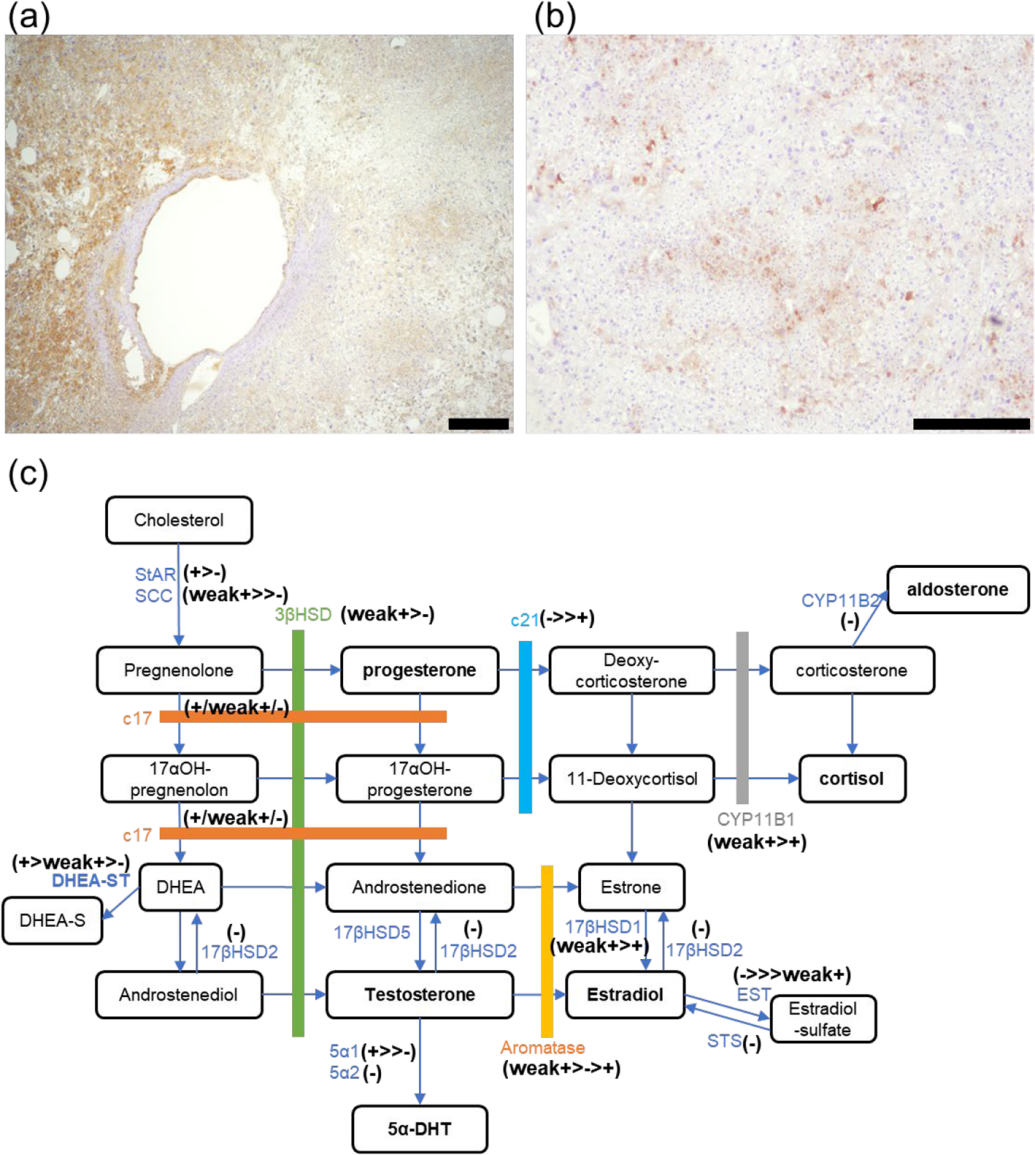
Immunohistochemical expressions of steroidogenic enzymes in the steroid cell tumor component. **a**, **b** Examples of heterogeneous expression of steroidogenic enzymes in steroid cell tumors. Immunohistochemistry for (**a**) aromatase and (**b**) c21. Bar=500 mm. **c** Summary of steroidogenesis pathway and the immunohistochemistry results of steroidogenic enzymes

The patient did not present with any clinical endocrine manifestations, and the endometrium was atrophic; therefore, serum hormone levels were not examined. We immunostained 15 steroidogenic enzymes (StAR, SCC, 3βHSD, CYP11B1, CYP11B2, c17, c21, 5α1, 5α2, aromatase, 17βHSD1, 17βHSD2, EST, SDS, and DHEA-ST) to further explore the types of steroid hormones produced in ovarian tumors. Steroidogenic enzymes besides CYP11B2, 17βHSD2, 5α2, and STS were expressed, but their immunoreactivity status was markedly heterogeneous, especially in steroid tumor cells with significant nuclear atypia (Fig. 2, Table 1). Fibroma cells showed weak and heterogeneous immunopositivity for steroidogenic enzymes, including 3βHSD, c17, 17βHSD1, 17βHSD2, aromatase, 5α1, and STS. SCT cells with less atypia exhibited immunostaining patterns similar to those of SCT cells with significant nuclear atypia, demonstrating immunopositivity for StAR, SCC, c17, and DHEA-ST, while being negative for CYP11B2 and 5α2. The immunoreactivity of 17βHSD2 and 5α1 in SCT cells with less atypia resembled that of fibroma cells, showing partial positivity for 17βHSD2 and weak positivity for 5α1. SCT cells at the metastatic site demonstrated intra-tumoral heterogeneity of steroidogenic enzymes, as observed in primary tumors (Table 1). The SCT cells were immunohistochemically positive for glucocorticoid receptor (GR) and progesterone receptor (PgR), and the fibroma component was positive for GR, PgR, and androgen receptor (AR).

**Fig. 3:**
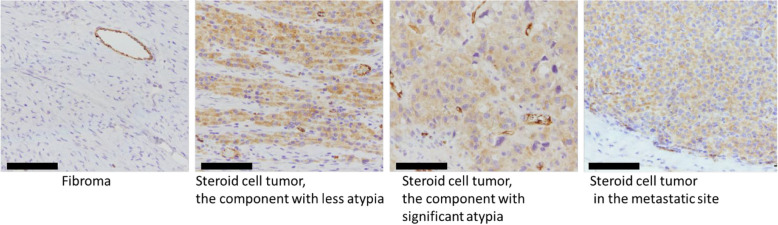
Immunohistochemistry of MUC4. Steroid cell tumor cells with/without significant atypia, including those of the metastatic site, were positive for MUC4, whereas fibroma cells were negative. Bar = 100 μm

Whole exome sequencing (WES) of these two tumor components, fibroma and SCTs, using FFPE specimens was performed to examine whether they were composite tumors derived from the same progenitor cell or collision tumors derived from different progenitor cells. No clinically significant or known pathogenic mutations known to be associated with malignancy were identified after filtering for potential single-nucleotide polymorphisms (SNPs). For example, both tumor components harbor a heterozygous missense mutation in *DICER1* (c.2033C > T); however, the pathogenic significance of this mutation remains unknown. Mutations in several genes, such as *ATRX*, *BAP1*, *BRCA2*, *CASP10*, *CDK4*, *CTNNB1*, *DCM1*, *FH*, *FOXO4*, *HIF1A*, *HOXA13*, *IDH2*, *LHCGR*, *MDM2*, *NPM1*, *SDHB*, *SRC*, *SS18*, *TP53*, and *VHL*, have been reported in SCT, NOS, and malignant cases [6–8]. However, no mutations in any of these genes were detected in our case. Immunohistochemical expressions of MLH1, MSH2, MSH6, and PMS2 were retained, and microsatellite instability was not demonstrated. Interestingly, six and four mutations in exon 2 of *MUC4* were identified in the SCT and fibroma components, respectively. Each tumor component harbored two missense variants, one of which was a common variant while the others were distinct (Table 2). Immunohistochemically, mucin 4 (MUC4) expression was observed in SCT cells, particularly in tumor cells with less atypia, including those at the metastatic site, whereas fibroma cells were immunonegative for MUC4 (Fig. 3). This may represent overexpression, as normal ovarian and adrenal gland cells showed weak positivity or were negative for MUC4 (Supplemental Fig. 1).

**Table 2:** MUC4 mutations

Steroid cell tumor		Fibroma	
p.Ser3221_Thr3268del	(Conservative inframe deletion)	p.Ala2425Gly	(Missense variant)
p.His2765His	(Synonymous variant)	p.Thr2255Thr	(Synonymous variant)
*p.Ser2055Phe*	(Missense variant)	*p.Ser2055Phe*	(Missense variant)
p.Ser1783Ser	(Synonymous variant)	p.Val1702Val	(Synonymous variant)
p.Ala1558Thr	(Missense variant)		
p.Thr1951_Pro1952insSerLeuProValThrAspAlaSerSerValSerThrGlyHisAlaThrSerLeuProValThrIleProSerSerAlaSerSerGlyHisThrThr	(Conservative inframe insertion)

## Discussions

Knowingly, this is the first reported case of an ovarian tumor with malignant SCT and fibroma components. A previously reported case of an ovarian tumor with SCT, NOS, and fibroma components demonstrated features similar to those in our case. The two components were macroscopically well demarcated, and the SCT component showed high mitotic activity and focal necrosis, which were reported to predict malignant behavior [4]. However, follow-up data of the patient were not reported in their study; therefore, whether any malignant behavior, such as recurrence or metastasis, was detected postoperatively remains unknown. The authors reported that the fibroma component contained aggregates of luteinized cells, which were not detected in our case, and postulated that the tumor was composite because the SCT component could have arisen from the neoplastic transformation of luteinized cells within the fibromatous component. However, there have been a few reports of fibromas with minor sex cord components [9] whose origin remains unclear. A tumor with two types of components is classified as either a composite or collision. Composite tumors typically share the same progenitor cells, whereas collision tumors arise from different progenitor cells. Morphologically, composite tumors have two distinct components that coexist, intermingled or with one predominant and a focal minority component [10]. Collision tumors comprise two well-circumscribed components. In our case, the fibroma and SCT components were macroscopically well-circumscribed, suggesting a collision tumor. However, microscopically, cuboidal steroid cells with fewer nuclear atypia were intermingled with fibroma cells at the boundary. Other sex cord components and potential progenitors of SCTs were not found in the fibroma. SCT cells with less atypia showed intermediate immunohistochemical features between those of fibromas and SCTs with marked nuclear atypia. Fibroma and SCTs harbored 621 and 779 single-nucleotide variants (SNVs) and indels, respectively. These two tumors shared 477 mutations, supporting a composite tumor diagnosis. However, a definitive differential diagnosis between collision or composite tumors could present diagnostic challenges because no previously reported driver mutations were detected by WES analysis. Missense variants of *MUC4* gene detected in SCT could contribute to the stabilization of the MUC4 protein or enhance its translation efficiency, although function of these variants is still unknown. MUC4 has been reported to be overexpressed in various tumors and to interact with ERBB2, promoting proliferation and contributing to malignancy [11]. MUC4 could also be associated with the development of malignant SCTs. The detailed pathogenesis of these tumors remains unclear. Therefore, investigations using animal models or in vitro models with stem cells, such as induced pluripotent stem cells, are warranted.

In this case, SCT exhibited disorganized expression patterns of steroidogenic enzymes, which are characteristic of adrenocortical cancers [12]. These tumors produce various hormones and precursors at different levels, leading to inefficient hormone receptor activation and a tendency to be non-functional. Here, the SCT shared features with adrenocortical carcinoma, including compact cells with diffuse growth, variable cytological atypia, mitosis, necrosis, and disorganized enzyme patterns. A comparative analysis not only between SCTs and other ovarian tumors but also with adrenal tumors may help elucidate the characteristics of malignant SCTs, warranting further investigation in the future. In addition, the fibroma and steroid cell components express the hormone receptors PgR, GR, and AR. Although a few efficient hormones may have been produced, these ligands may have only weakly enhanced the growth of both components. The impact of steroid hormones on tumor development and growth should also be taken into consideration.

## Conclusion

This case of an ovarian tumor comprising fibromas and malignant SCT components highlights the complexity of sex cord-stromal tumors, particularly the mechanisms underlying the development and malignant transformation of SCTs. Although immunohistochemistry and genetic analyses were performed in this case, these mechanisms remain unclear. Further case studies are required to advance our understanding of these tumors.

## Supplementary Information

Supplementary Material 1: Supplemental Figure 1. Normal ovarian granulosa and stromal cell, and adrenal gland cells showed weak positivity or negativity for MUC4. Bar = 100 µm.

Supplementary Material 2. Supplement: materials and methods of whole-exome sequencing (WES). DNA was extracted from the fibroma and steroid cell tumor tissues using the QIAamp DNA FFPE Tissue Kit (Qiagen, Valencia, CA, USA) according to the manufacturer’s protocol. Extracted genomic DNA was subjected to WES. WES was outsourced to Macrogen Japan Company and performed using the SureSelect V6-Post and Illumina platforms. Paired-end sequences produced by the NovaSeq Instrument were mapped to the human reference gene GRCh38 using the mapping program BWA, and variant calling was performed using GATK. Mutations with variant allele frequency < 0.1, depth < 30, or those registered in 1000 Genomes with AF > 0.05 (possible SNPs) were excluded for filtering germline mutations.

## Data Availability

The data supporting the findings of this case report are not publicly available due to sensitivity concerns but can be obtained from the corresponding author upon reasonable request.

## Abbreviations

SCT: Steroid cell tumor
FIGO: International Federation of Gynecology and Obstetrics
NOS: Not otherwise specified
MRI: Magnetic resonance imaging
StAR: Steroidogenic acute regulatory protein
SCC: Side-chain cleavage enzyme
3βHSD: 3β-Hydroxysteroid dehydrogenase
CYP11B1: Cytochrome P450 family 11 subfamily B member 1
CYP11B2: Cytochrome P450 family 11 subfamily B member 2
C17: 17α-Hydroxylase
C21: 21-Hydroxylase
5α1: 5α-Reductase type 1
5α2: 5α-Reductase type 2
Aromatase: Cytochrome P450 family 19 subfamily A member 1
17βHSD1: 17β-Hydroxysteroid dehydrogenase type 1
17βHSD2: 17β-Hydroxysteroid dehydrogenase type 2
EST: Estrone sulfotransferase
SDS: Steroid sulfatase
DHEA-ST: Dehydroepiandrosterone sulfotransferase
GR: Glucocorticoid receptor
PgR: Progesterone receptor
AR: Androgen receptor
WES: Whole exome sequencing
SNP: Single-nucleotide polymorphisms
MUC4: Mucin 4
SNV: Single-nucleotide variants
