# Supplementary material for: A rare case of a concomitant ovarian fibroma and malignant steroid cell tumor: insights into pathogenesis and steroidogenesis
Source: J Egypt Natl Canc Inst. 2025 May 19;37:21. doi: 10.1186/s43046-025-00281-3 (PMC13313433; doi:10.1186/s43046-025-00281-3)
Supplement: Supplementary file 1 — Supplementary Material 1: Supplemental Figure 1. Normal ovarian granulosa and stromal cell, and adrenal gland cells showed weak positivity or negativity for MUC4. Bar = 100 µm. [file 43046_2025_281_MOESM1_ESM.pptx]

## Slide 1
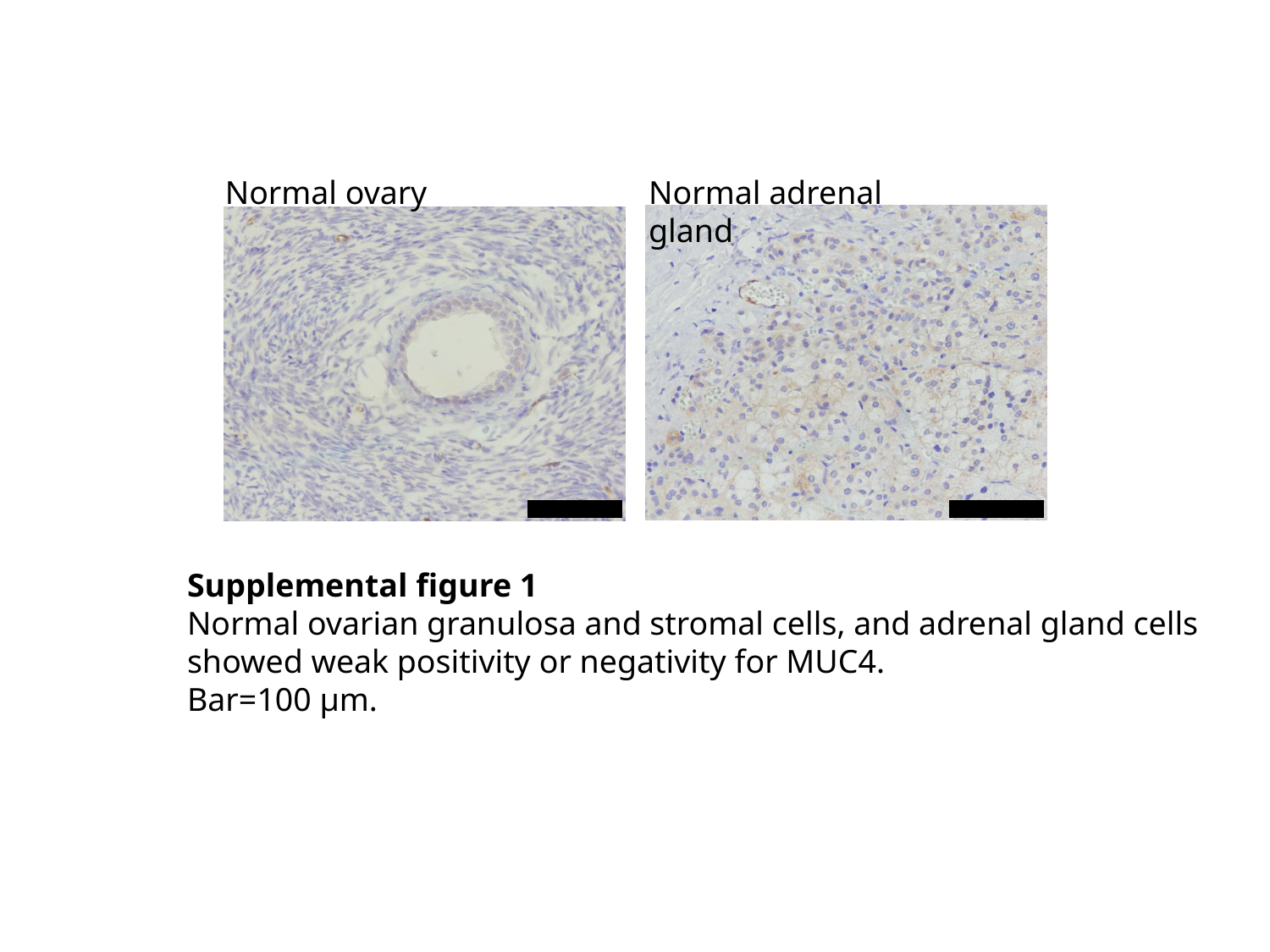

Normal ovary
Normal adrenal gland
Supplemental figure 1
Normal ovarian granulosa and stromal cells, and adrenal gland cells
showed weak positivity or negativity for MUC4.
Bar=100 μm.
