# Supplementary material for: A rare case of a concomitant ovarian fibroma and malignant steroid cell tumor: insights into pathogenesis and steroidogenesis
Source: J Egypt Natl Canc Inst. 2025 May 19;37:21. doi: 10.1186/s43046-025-00281-3 (PMC13313433; doi:10.1186/s43046-025-00281-3)
Supplement: Supplementary file 2 — Supplementary Material 2. Supplement: materials and methods of whole-exome sequencing (WES). DNA was extracted from the fibroma and steroid cell tumor tissues using the QIAamp DNA FFPE Tissue Kit (Qiagen, Valencia, CA, USA) according to the manufacturer’s protocol. Extracted genomic DNA was subjected to WES. WES was outsourced to Macrogen Japan Company and performed using the SureSelect V6-Post and Illumina platforms. Paired-end sequences produced by the NovaSeq Instrument were mapped to the human reference gene GRCh38 using the mapping program BWA, and variant calling was performed using GATK. Mutations with variant allele frequency < 0.1, depth < 30, or those registered in 1000 Genomes with AF > 0.05 (possible SNPs) were excluded for filtering germline mutations. [file 43046_2025_281_MOESM2_ESM.docx]

Supplement: materials and methods of whole-exome sequencing (WES)

DNA was extracted from the fibroma and steroid cell tumor tissues using the QIAamp DNA FFPE Tissue Kit (Qiagen, Valencia, CA, USA) according to the manufacturer’s protocol. Extracted genomic DNA was subjected to WES. WES was outsourced to Macrogen Japan Company and performed using the SureSelect V6-Post and Illumina platforms. Paired-end sequences produced by the NovaSeq Instrument were mapped to the human reference gene GRCh38 using the mapping program BWA, and variant calling was performed using GATK. Mutations with variant allele frequency < 0.1, depth < 30, or those registered in 1000 Genomes with AF > 0.05 (possible SNPs) were excluded for filtering germline mutations.
